# *N*′-[(*E*)-5-Bromo-2-hy­droxy-3-meth­oxy­benzyl­idene]benzohydrazide monohydrate

**DOI:** 10.1107/S1600536813030572

**Published:** 2013-11-16

**Authors:** Jessy Emmanuel, M. Sithambaresan, M. R. Prathapachandra Kurup

**Affiliations:** a Department of Chemistry, St. Joseph’s College, Irinjalakuda, India; b Department of Chemistry, Faculty of Science, Eastern University, Sri Lanka, Chenkalady, Sri Lanka; c Department of Applied Chemistry, Cochin University of Science and Technology, Kochi 682 022, India

## Abstract

The title compound, C_15_H_13_BrN_2_O_3_·H_2_O, exists in an *E* conformation with respect to the azo­methane C=N double bond. The benzene and phenyl rings form dihedral angles of 0.46 (2) and 4.90 (3)°, respectively with the central C(=O)N_2_C unit. An intra­molecular O—H⋯N hydrogen bond occurs. In the crystal, some hydrazide mol­ecules are replaced by mol­ecules of the 6-bromo isomer. The Br atom from this admixture was refined to give a partial occupancy of 0.0443 (19). A supra­molecular network is built in the lattice by means of inter­molecular N—H⋯O and two O—H⋯O inter­actions together with non-classical C—H⋯O inter­actions involving the lattice water mol­ecule stacking the mol­ecules along the *b-*axis direction.

## Related literature

For biological applications of benzohydrazones and derivatives, see: Sreeja *et al.* (2004 ▶); Rada & Leto (2008 ▶); Rakha *et al.* (1996 ▶); Takahama (1996 ▶). For the synthesis of related compounds, see: Emmanuel *et al.* (2011 ▶). For a related structure, see Reshma *et al.* (2012 ▶).
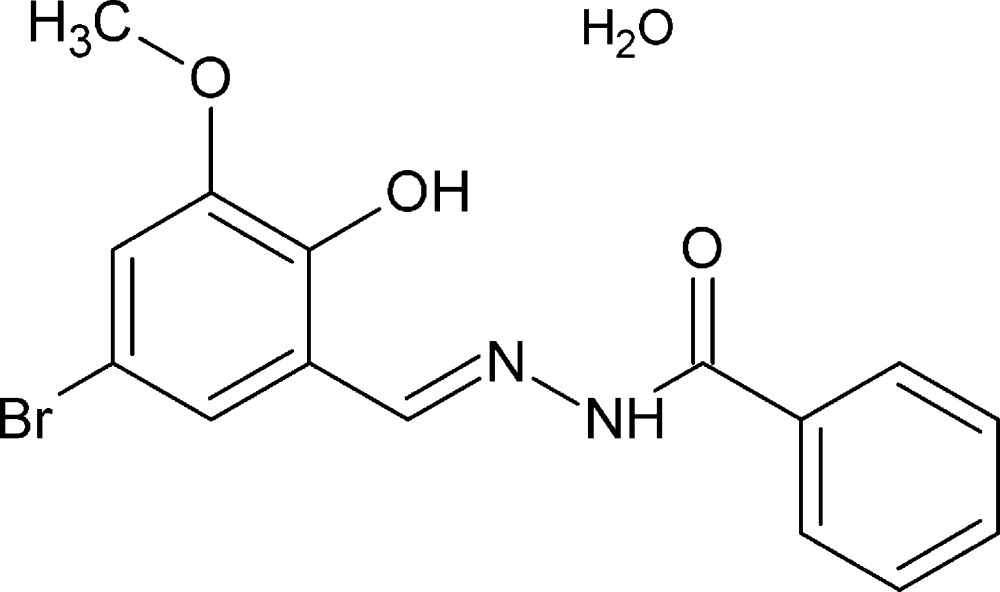


## Experimental

### 

#### Crystal data

C_15_H_13_BrN_2_O_3_·H_2_O
*M* *_r_* = 367.20
Orthorhombic, 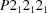

*a* = 4.7223 (5) Å
*b* = 13.9357 (17) Å
*c* = 23.028 (3) Å
*V* = 1515.4 (3) Å^3^
*Z* = 4
Mo *K*α radiation
μ = 2.73 mm^−1^
*T* = 293 K
0.32 × 0.25 × 0.22 mm

#### Data collection

Bruker Kappa APEXII CCD diffractometer
Absorption correction: multi-scan (*SADABS*; Bruker, 2004 ▶) *T*_min_ = 0.446, *T*_max_ = 0.549
12759 measured reflections
2966 independent reflections
2189 reflections with *I* > 2σ(*I*)
*R*_int_ = 0.090

#### Refinement

*R*[*F* ^2^ > 2σ(*F* ^2^)] = 0.044
*wR*(*F* ^2^) = 0.114
*S* = 0.89
2966 reflections
218 parameters
7 restraints
H atoms treated by a mixture of independent and constrained refinement
Δρ_max_ = 0.45 e Å^−3^
Δρ_min_ = −0.29 e Å^−3^
Absolute structure: Flack (1983 ▶), 1203 Friedel pairs
Absolute structure parameter: 0.016 (16)

### 

Data collection: *APEX2* (Bruker, 2004 ▶); cell refinement: *APEX2* and *SAINT* (Bruker, 2004 ▶); data reduction: *SAINT* and *XPREP* (Bruker, 2004 ▶); program(s) used to solve structure: *SHELXS97* (Sheldrick, 2008 ▶); program(s) used to refine structure: *SHELXL97* (Sheldrick, 2008 ▶); molecular graphics: *ORTEP-3 for Windows* (Farrugia, 2012 ▶) and *DIAMOND* (Brandenburg, 2010 ▶); software used to prepare material for publication: *SHELXL97* and *publCIF* (Westrip, 2010 ▶).

## Supplementary Material

Crystal structure: contains datablock(s) Global, I. DOI: 10.1107/S1600536813030572/hg5359sup1.cif

Structure factors: contains datablock(s) I. DOI: 10.1107/S1600536813030572/hg5359Isup2.hkl

Supplementary material file. DOI: 10.1107/S1600536813030572/hg5359Isup3.cml

Additional supplementary materials:  crystallographic information; 3D view; checkCIF report

## Figures and Tables

**Table 1:** Hydrogen-bond geometry (Å, °)

*D*—H⋯*A*	*D*—H	H⋯*A*	*D*⋯*A*	*D*—H⋯*A*
N2—H2⋯O1*W* ^i^	0.86	2.11	2.946 (5)	163
O1—H1⋯N1	0.83	1.93	2.637 (5)	142
O1*W*—H1*B*⋯O2^ii^	0.86 (2)	2.50 (5)	3.178 (5)	136 (6)
O1*W*—H1*B*⋯O1^ii^	0.86 (2)	2.27 (4)	3.051 (5)	151 (6)
O1*W*—H1*A*⋯O3	0.86 (2)	1.91 (3)	2.736 (5)	163 (6)
C7—H7⋯O1*W* ^i^	0.93	2.50	3.305 (6)	145
C10—H10⋯O1*W* ^i^	0.93	2.42	3.329 (6)	166
C11—H11⋯O2^i^	0.93	2.55	3.435 (5)	160

Symmetry codes: (i) 
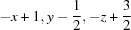
; (ii) 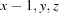.

**Fig. 1.**
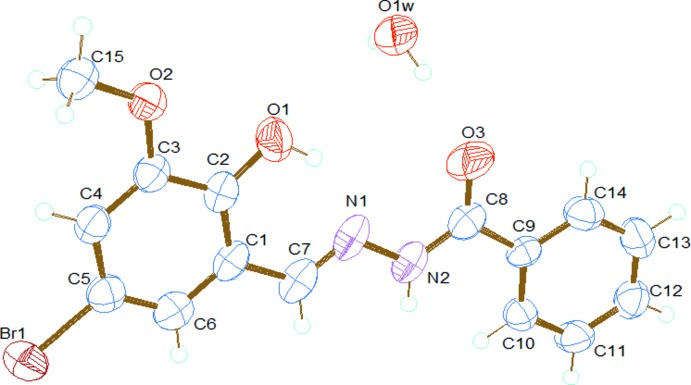
ORTEP view of the title compound drawn with 50% probability displacement ellipsoids for the non-H atoms. Bromine and the hydrogen atoms of the admixture was omitted.

**Fig. 2.**
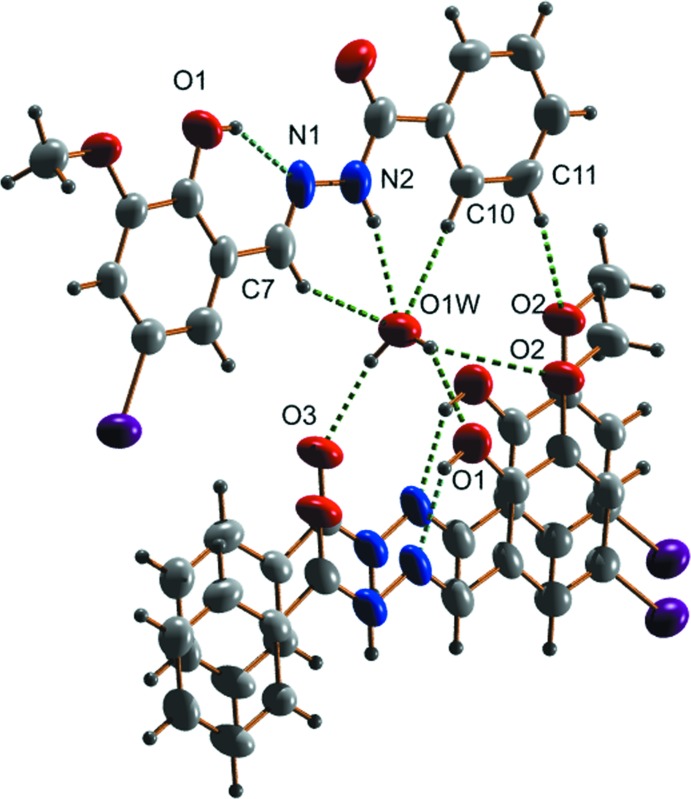
Hydrogen-bonding interactions in the crystal structure of C15H13BrN2O3·H2O.

**Fig. 3.**
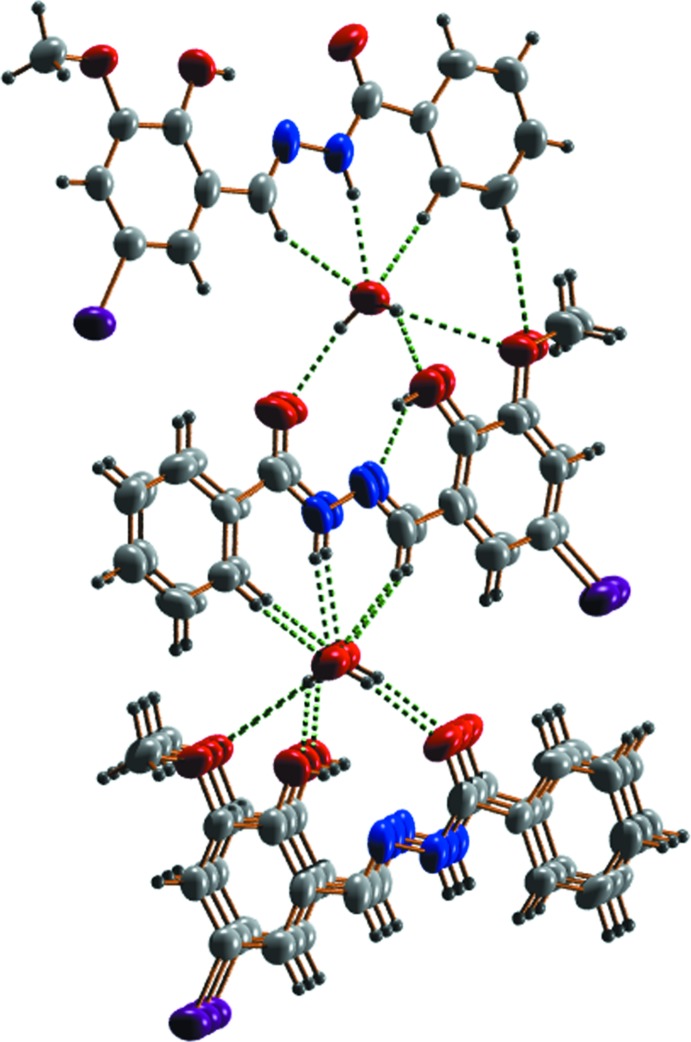
Molecules are stacked one over the other by means of intermolecular hydrogen bonding interactions in the crystal structure of the title compound.

**Fig. 4.**
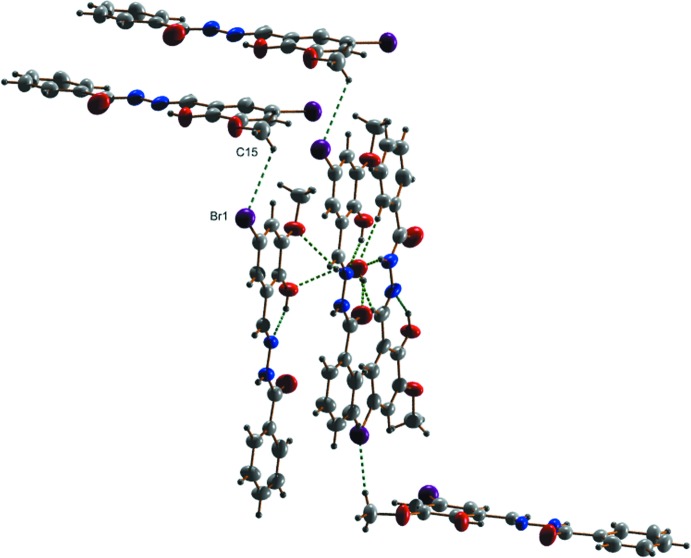
C–H···Br interactions interconnecting the two one-dimensional-layers in the lattice.

**Fig. 5.**
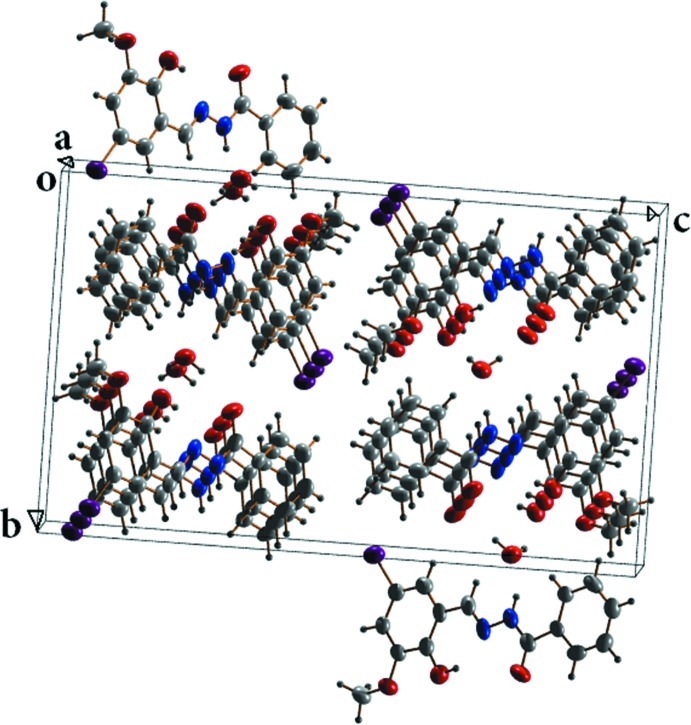
Packing diagram of the compound along the a axis.

## supplementary crystallographic information

### 1. Comment

Hydrazone derivatives represent an important class of organic compounds. The research for this class of compounds is an area of great interest due to their biological activities (Sreeja *et al.*, 2004; Rada & Leto, 2008). They have been extensively investigated recently owing to their potential application as antineoplastic, antiviral and antiinflammatory agents (Rakha *et al.*, 1996; Takahama, 1996).

The compound (Fig. 1) crystallizes in the monoclinic space group *P*2_1_2_1_2_1_. This molecule adopts an *E* configuration with respect to the C7=N1 bond and it exists in the amido form with a C8=O3 bond length of 1.222 (5) Å which is very close to the reported C=O bond length of a related structure (Reshma *et al.*, 2012). The O3 and N1 atoms are in a *Z* configuration with respect to C8–N2 having a torsion angle of -0.3 (7)°. The central C(=O)N_2_C unit has dihedral angles of 0.46 (2) and 4.90 (3)°, respectively with the phenol and phenyl rings.

In the crystal, approximately 4% of the title compound is replaced by molecules of the 6-isomer, the Br1B atom of this admixture molecule was included in the refinement. Since the molecules of the 6-isomer are likely to be non-planar due to sterical factors, it does not occupy the same position as the molecule of the 5-bromo isomer. As a result, Br1B deviates by 0.39 (2) Å from the mean plane of C1—C6 plane, and the distance C6—Br1B is 1.798 (8) Å, much smaller than the typical bond length of C—Br.

The lattice water molecule connects three adjacent molecules *via* three classical O–H···O and a N–H···O hydrogen bond interactions with D···A distances of 3.178 (5), 3.052 (5), 2.736 (5) and 2.945 (5) Å and two non-classical C–H···O hydrogen bond interactions with D···A distances of 3.304 (8) and 3.333 (7) Å (Fig. 2, Table 1). Molecules are stacked one over the other by forming a one-dimensional-layer *via* O–H···O, N–H···O and C–H···O intermolecular hydrogen bonding along *a* axis (Fig. 3). Such layers are connected by means of a C–H···Br intermolecular hydrogen bonding interaction with D···A distance of 3.649 (5) Å (Fig. 4). These layers arranged in a zig-zag fashion (Fig. 4) forming a three-dimensional-supramolecular network in the lattice. The molecule also has a O–H···N intramolecular hydrogen bonding with a D···A distance of 2.637 (5) Å. Although there are very few weak short ring interactions found in the crystal system, they are not significant to support the network since centroid-centroid distances are above 4 Å. Fig. 5 shows a packing diagram of the title compound viewed along the *a* axis.

### 2. Experimental

The title compound was prepared by adapting a reported procedure (Emmanuel *et al.*, 2011). A solution of 5-bromo-3-methoxysalicylaldehyde (0.231 g, 1 mmol) in ethanol (10 ml) was mixed with an ethanolic solution (10 ml) of benzhydrazide (0.228 g, 1 mmol). The mixture was boiled under reflux for 3 h and then cooled to room temperature. The formed product was recrystallized in ethanol, washed with few drops of ethanol and dried over P_4_O_10_ in *vacuo*. Colorless block shaped crystals, suitable for single-crystal XRD studies, were obtained after slow evaporation of the solution in air for a few days.

### 3. Refinement

The bromine atoms Br1 and Br1B of this molecule were refined freely, with the sum of their occupancy factors constrained to 1.0. The atoms H2, Br1B, H1A and H1B were located from a difference Fourier map and N2—H2 distance was restrained to 0.88±0.02. The H5 atom was placed in calculated position with occupancy factor equal to that Br1B, and its coordinates were fixed. The H6 atom was refined with restrained distance of 0.93 with occupancy factor equal to that of Br1. O1W–H1A and O1W–H1B distances were restrained to 0.85±0.02. C6–Br1B distance is restrained to 1.88±0.01 Å. The H atoms on C were placed in calculated positions, guided by difference maps, with C–H bond distances 0.93–0.96 Å. H atoms were assigned as *U*_iso_(H) = 1.2U_eq_(carrier) or 1.5U_eq_ (methyl C). Omitted owing to bad disagreement were the reflections (0 0 2) and (0 1 1).

### Crystal data

**Table:**

C_15_H_13_BrN_2_O_3_·H_2_O	*F*(000) = 744
*M**_r_* = 367.20	*D*_x_ = 1.609 Mg m^−^^3^
Orthorhombic, *P*2_1_2_1_2_1_	Mo *K*α radiation, λ = 0.71073 Å
Hall symbol: P 2ac 2ab	Cell parameters from 3233 reflections
*a* = 4.7223 (5) Å	θ = 2.3–28.0°
*b* = 13.9357 (17) Å	µ = 2.73 mm^−^^1^
*c* = 23.028 (3) Å	*T* = 293 K
*V* = 1515.4 (3) Å^3^	Block, pale brown
*Z* = 4	0.32 × 0.25 × 0.22 mm

### Data collection

**Table:**

Bruker Kappa APEXII CCD diffractometer	2966 independent reflections
Radiation source: fine-focus sealed tube	2189 reflections with *I* > 2σ(*I*)
Graphite monochromator	*R*_int_ = 0.090
Detector resolution: 8.33 pixels mm^-1^	θ_max_ = 26.0°, θ_min_ = 2.9°
ω and φ scan	*h* = −5→5
Absorption correction: multi-scan (*SADABS*; Bruker, 2004)	*k* = −17→17
*T*_min_ = 0.446, *T*_max_ = 0.549	*l* = −22→28
12759 measured reflections	

### Refinement

**Table:**

Refinement on *F*^2^	Secondary atom site location: difference Fourier map
Least-squares matrix: full	Hydrogen site location: inferred from neighbouring sites
*R*[*F*^2^ > 2σ(*F*^2^)] = 0.044	H atoms treated by a mixture of independent and constrained refinement
*wR*(*F*^2^) = 0.114	*w* = 1/[σ^2^(*F*_o_^2^) + (0.*P*)^2^ + 1.2809*P*] where *P* = (*F*_o_^2^ + 2*F*_c_^2^)/3
*S* = 0.89	(Δ/σ)_max_ = 0.001
2966 reflections	Δρ_max_ = 0.45 e Å^−^^3^
218 parameters	Δρ_min_ = −0.29 e Å^−^^3^
7 restraints	Absolute structure: Flack (1983), 1203 Friedel pairs
Primary atom site location: structure-invariant direct methods	Absolute structure parameter: 0.016 (16)

### Special details

**Table:**

Geometry. All e.s.d.'s (except the e.s.d. in the dihedral angle between two l.s. planes) are estimated using the full covariance matrix. The cell e.s.d.'s are taken into account individually in the estimation of e.s.d.'s in distances, angles and torsion angles; correlations between e.s.d.'s in cell parameters are only used when they are defined by crystal symmetry. An approximate (isotropic) treatment of cell e.s.d.'s is used for estimating e.s.d.'s involving l.s. planes.
Refinement. Refinement of *F*^2^ against ALL reflections. The weighted *R*-factor *wR* and goodness of fit *S* are based on *F*^2^, conventional *R*-factors *R* are based on *F*, with *F* set to zero for negative *F*^2^. The threshold expression of *F*^2^ > σ(*F*^2^) is used only for calculating *R*-factors(gt) *etc*. and is not relevant to the choice of reflections for refinement. *R*-factors based on *F*^2^ are statistically about twice as large as those based on *F*, and *R*- factors based on ALL data will be even larger.

### Fractional atomic coordinates and isotropic or equivalent isotropic displacement parameters (Å^2^)

**Table:**

	*x*	*y*	*z*	*U*_iso_*/*U*_eq_	Occ. (<1)
Br1	1.56048 (13)	0.50757 (4)	0.94230 (3)	0.0664 (2)	0.956 (2)
Br1B	1.181 (4)	0.4776 (7)	0.8289 (6)	0.087 (7)	0.044 (2)
O1	1.0006 (7)	0.8426 (2)	0.82729 (16)	0.0610 (10)	
H1	0.906 (14)	0.8268 (14)	0.798 (3)	0.092*	
O2	1.3713 (8)	0.8739 (2)	0.90679 (15)	0.0598 (9)	
O3	0.4122 (11)	0.8432 (2)	0.70096 (17)	0.0800 (12)	
N1	0.7166 (9)	0.7183 (4)	0.76351 (16)	0.0552 (11)	
N2	0.5260 (8)	0.6904 (3)	0.72170 (16)	0.0536 (11)	
H2	0.4997	0.6305	0.7146	0.064*	
C2	1.1208 (10)	0.7645 (3)	0.85060 (19)	0.0479 (11)	
C3	1.3235 (10)	0.7802 (3)	0.8941 (2)	0.0478 (11)	
C4	1.4551 (10)	0.7037 (3)	0.92142 (19)	0.0467 (11)	
H4	1.5857	0.7140	0.9510	0.056*	
C5	1.3871 (10)	0.6112 (3)	0.9036 (2)	0.0496 (11)	
H5	1.4744	0.5592	0.9214	0.060*	0.044 (2)
C6	1.1964 (11)	0.5953 (3)	0.8607 (2)	0.0527 (12)	
H6	1.1570	0.5327	0.8492	0.063*	0.956 (2)
C1	1.0590 (11)	0.6710 (3)	0.83351 (19)	0.0479 (11)	
C7	0.8523 (11)	0.6512 (4)	0.7890 (2)	0.0555 (13)	
H7	0.8168	0.5879	0.7787	0.067*	
C8	0.3806 (11)	0.7575 (3)	0.6918 (2)	0.0516 (12)	
C9	0.1805 (10)	0.7211 (3)	0.64786 (18)	0.0419 (10)	
C10	0.1193 (10)	0.6254 (3)	0.6384 (2)	0.0489 (12)	
H10	0.2073	0.5787	0.6609	0.059*	
C11	−0.0706 (12)	0.5983 (3)	0.5959 (2)	0.0587 (13)	
H11	−0.1095	0.5336	0.5901	0.070*	
C12	−0.2013 (12)	0.6656 (4)	0.5626 (2)	0.0595 (13)	
H12	−0.3291	0.6470	0.5340	0.071*	
C13	−0.1445 (12)	0.7607 (4)	0.5713 (2)	0.0603 (14)	
H13	−0.2330	0.8068	0.5484	0.072*	
C14	0.0435 (11)	0.7882 (3)	0.6138 (2)	0.0559 (12)	
H14	0.0791	0.8531	0.6196	0.067*	
C15	1.5878 (12)	0.8956 (3)	0.9469 (2)	0.0616 (13)	
H15A	1.5379	0.8706	0.9844	0.092*	
H15B	1.6109	0.9639	0.9494	0.092*	
H15C	1.7621	0.8669	0.9343	0.092*	
O1W	0.4629 (10)	0.9794 (2)	0.78591 (18)	0.0782 (11)	
H1A	0.473 (15)	0.944 (4)	0.7555 (18)	0.117*	
H1B	0.346 (12)	0.952 (5)	0.809 (2)	0.117*	

### Atomic displacement parameters (Å^2^)

**Table:**

	*U*^11^	*U*^22^	*U*^33^	*U*^12^	*U*^13^	*U*^23^
Br1	0.0768 (4)	0.0492 (3)	0.0733 (4)	0.0101 (3)	−0.0021 (3)	0.0056 (3)
Br1B	0.127 (13)	0.058 (8)	0.077 (10)	−0.019 (8)	0.028 (9)	0.004 (7)
O1	0.059 (3)	0.064 (2)	0.060 (2)	0.0015 (17)	−0.0188 (19)	0.0050 (17)
O2	0.064 (2)	0.0475 (18)	0.067 (2)	0.0032 (17)	−0.0247 (19)	−0.0072 (16)
O3	0.108 (3)	0.054 (2)	0.078 (3)	−0.020 (2)	−0.012 (3)	−0.0184 (19)
N1	0.048 (2)	0.081 (3)	0.037 (2)	−0.018 (2)	0.000 (2)	−0.010 (2)
N2	0.051 (3)	0.068 (2)	0.042 (2)	−0.012 (2)	−0.003 (2)	−0.009 (2)
C2	0.045 (3)	0.059 (3)	0.039 (2)	−0.003 (2)	−0.001 (2)	−0.002 (2)
C3	0.049 (3)	0.049 (3)	0.045 (3)	−0.003 (2)	−0.005 (2)	−0.005 (2)
C4	0.044 (2)	0.055 (2)	0.041 (2)	0.002 (2)	−0.001 (2)	−0.005 (2)
C5	0.050 (3)	0.052 (3)	0.046 (3)	0.000 (2)	0.005 (2)	−0.001 (2)
C6	0.056 (3)	0.052 (3)	0.050 (3)	−0.006 (2)	0.004 (3)	−0.004 (2)
C1	0.044 (2)	0.063 (3)	0.036 (2)	−0.008 (3)	0.004 (2)	−0.009 (2)
C7	0.049 (3)	0.076 (3)	0.042 (3)	−0.015 (3)	0.004 (2)	−0.010 (3)
C8	0.054 (3)	0.057 (3)	0.044 (3)	−0.007 (3)	0.006 (2)	−0.008 (2)
C9	0.043 (2)	0.044 (2)	0.039 (2)	−0.002 (2)	0.007 (2)	−0.007 (2)
C10	0.053 (3)	0.042 (2)	0.052 (3)	0.001 (2)	−0.010 (2)	−0.002 (2)
C11	0.062 (3)	0.047 (3)	0.067 (3)	0.003 (3)	−0.013 (3)	−0.015 (2)
C12	0.057 (3)	0.069 (3)	0.052 (3)	0.006 (3)	−0.009 (3)	−0.005 (3)
C13	0.061 (3)	0.063 (3)	0.057 (3)	0.008 (3)	−0.006 (3)	0.011 (3)
C14	0.063 (3)	0.043 (2)	0.061 (3)	−0.002 (3)	0.005 (3)	0.000 (2)
C15	0.061 (3)	0.059 (3)	0.065 (3)	−0.002 (3)	−0.017 (3)	−0.011 (3)
O1W	0.111 (3)	0.053 (2)	0.071 (2)	−0.012 (2)	−0.018 (2)	−0.0054 (18)

### Geometric parameters (Å, º)

**Table:**

Br1—C5	1.885 (5)	C1—C7	1.441 (7)
Br1B—C6	1.798 (8)	C7—H7	0.9300
O1—C2	1.339 (6)	C8—C9	1.475 (6)
O1—H1	0.8311	C9—C14	1.382 (6)
O2—C3	1.357 (5)	C9—C10	1.382 (6)
O2—C15	1.411 (6)	C10—C11	1.379 (7)
O3—C8	1.221 (5)	C10—H10	0.9300
N1—C7	1.277 (7)	C11—C12	1.360 (7)
N1—N2	1.374 (5)	C11—H11	0.9300
N2—C8	1.349 (6)	C12—C13	1.366 (7)
N2—H2	0.8600	C12—H12	0.9300
C2—C1	1.392 (6)	C13—C14	1.376 (7)
C2—C3	1.403 (6)	C13—H13	0.9300
C3—C4	1.385 (6)	C14—H14	0.9300
C4—C5	1.391 (6)	C15—H15A	0.9600
C4—H4	0.9300	C15—H15B	0.9600
C5—C6	1.355 (7)	C15—H15C	0.9600
C5—H5	0.9300	O1W—H1A	0.86 (2)
C6—C1	1.387 (6)	O1W—H1B	0.86 (2)
C6—H6	0.9300		
			
C2—O1—H1	109.5	C1—C7—H7	119.1
C3—O2—C15	117.9 (4)	O3—C8—N2	121.9 (5)
C7—N1—N2	116.3 (5)	O3—C8—C9	122.2 (5)
C8—N2—N1	119.6 (4)	N2—C8—C9	116.0 (4)
C8—N2—H2	120.2	C14—C9—C10	117.7 (4)
N1—N2—H2	120.2	C14—C9—C8	117.2 (4)
O1—C2—C1	123.9 (4)	C10—C9—C8	125.1 (4)
O1—C2—C3	116.7 (4)	C11—C10—C9	120.8 (4)
C1—C2—C3	119.4 (4)	C11—C10—H10	119.6
O2—C3—C4	124.6 (4)	C9—C10—H10	119.6
O2—C3—C2	114.6 (4)	C12—C11—C10	120.4 (4)
C4—C3—C2	120.7 (4)	C12—C11—H11	119.8
C3—C4—C5	118.4 (4)	C10—C11—H11	119.8
C3—C4—H4	120.8	C11—C12—C13	119.8 (5)
C5—C4—H4	120.8	C11—C12—H12	120.1
C6—C5—C4	121.4 (4)	C13—C12—H12	120.1
C6—C5—Br1	120.6 (4)	C12—C13—C14	120.1 (5)
C4—C5—Br1	118.0 (4)	C12—C13—H13	120.0
C6—C5—H5	119.3	C14—C13—H13	120.0
C4—C5—H5	119.3	C9—C14—C13	121.2 (4)
C5—C6—C1	121.0 (4)	C9—C14—H14	119.4
C5—C6—Br1B	118.3 (7)	C13—C14—H14	119.4
C1—C6—Br1B	119.4 (7)	O2—C15—H15A	109.5
C5—C6—H6	119.5	O2—C15—H15B	109.5
C1—C6—H6	119.5	H15A—C15—H15B	109.5
C6—C1—C2	119.1 (4)	O2—C15—H15C	109.5
C6—C1—C7	119.4 (5)	H15A—C15—H15C	109.5
C2—C1—C7	121.5 (5)	H15B—C15—H15C	109.5
N1—C7—C1	121.8 (5)	H1A—O1W—H1B	106 (4)
N1—C7—H7	119.1		
			
C7—N1—N2—C8	−178.2 (4)	C3—C2—C1—C6	1.1 (7)
C15—O2—C3—C4	5.9 (7)	O1—C2—C1—C7	−1.1 (7)
C15—O2—C3—C2	−175.2 (4)	C3—C2—C1—C7	180.0 (4)
O1—C2—C3—O2	−0.1 (6)	N2—N1—C7—C1	179.7 (4)
C1—C2—C3—O2	178.9 (4)	C6—C1—C7—N1	178.7 (5)
O1—C2—C3—C4	178.8 (4)	C2—C1—C7—N1	−0.2 (7)
C1—C2—C3—C4	−2.2 (7)	N1—N2—C8—O3	−0.3 (7)
O2—C3—C4—C5	−179.5 (5)	N1—N2—C8—C9	−179.9 (4)
C2—C3—C4—C5	1.8 (7)	O3—C8—C9—C14	4.8 (7)
C3—C4—C5—C6	−0.2 (7)	N2—C8—C9—C14	−175.5 (4)
C3—C4—C5—Br1	−178.5 (4)	O3—C8—C9—C10	−175.0 (5)
C4—C5—C6—C1	−0.9 (7)	N2—C8—C9—C10	4.7 (7)
Br1—C5—C6—C1	177.3 (4)	C14—C9—C10—C11	0.5 (7)
C4—C5—C6—Br1B	165.9 (7)	C8—C9—C10—C11	−179.7 (5)
Br1—C5—C6—Br1B	−15.9 (8)	C9—C10—C11—C12	0.0 (8)
C5—C6—C1—C2	0.5 (7)	C10—C11—C12—C13	−0.1 (9)
Br1B—C6—C1—C2	−166.1 (7)	C11—C12—C13—C14	−0.3 (9)
C5—C6—C1—C7	−178.5 (4)	C10—C9—C14—C13	−0.8 (7)
Br1B—C6—C1—C7	14.9 (9)	C8—C9—C14—C13	179.4 (5)
O1—C2—C1—C6	−180.0 (4)	C12—C13—C14—C9	0.8 (8)

### Hydrogen-bond geometry (Å, º)

**Table:**

*D*—H···*A*	*D*—H	H···*A*	*D*···*A*	*D*—H···*A*
N2—H2···O1*W*^i^	0.86	2.11	2.946 (5)	163
O1—H1···N1	0.83	1.93	2.637 (5)	142
O1*W*—H1*B*···O2^ii^	0.86 (2)	2.50 (5)	3.178 (5)	136 (6)
O1*W*—H1*B*···O1^ii^	0.86 (2)	2.27 (4)	3.051 (5)	151 (6)
O1*W*—H1*A*···O3	0.86 (2)	1.91 (3)	2.736 (5)	163 (6)
C7—H7···O1*W*^i^	0.93	2.50	3.305 (6)	145
C10—H10···O1*W*^i^	0.93	2.42	3.329 (6)	166
C11—H11···O2^i^	0.93	2.55	3.435 (5)	160

Symmetry codes: (i) −*x*+1, *y*−1/2, −*z*+3/2; (ii) *x*−1, *y*, *z*.
